# Early Loading of Two Implants Supporting Mandibular Overdentures in Geriatric Edentulous Patients: A 12-Year Follow-Up Study

**DOI:** 10.3390/jcm12113825

**Published:** 2023-06-02

**Authors:** Eugenio Velasco-Ortega, Nuno Matos-Garrido, Alvaro Jiménez-Guerra, Ivan Ortiz-Garcia, Jesús Moreno-Muñoz, Enrique Núñez-Márquez, José Luis Rondón-Romero, Raul Ayuso-Montero, José López-López, Loreto Monsalve-Guil

**Affiliations:** 1 Comprehensive Dentistry for Adults and Gerodontology, Faculty of Dentistry, University of Seville, 41004 Sevilla, Spain; evelasco@us.es (E.V.-O.); nunogarrido@orallagos.pt (N.M.-G.); ivanortizgarcia1000@hotmail.com (I.O.-G.); je5us@hotmail.com (J.M.-M.); enrique_aracena@hotmail.com (E.N.-M.); jolurr001@hotmail.com (J.L.R.-R.); lomonsalve@hotmail.es (L.M.-G.); 2 Department of Odontostomatology, Prosthodontics, Occlusion and Oral Rehabilitation Unit, Faculty of Medicine and Health Sciences, University of Barcelona, 08907 Barcelona, Spain; raulayuso@ub.edu; 3 Department of Odontostomatology, Oral Medicine Unit, Faculty of Medicine and Health Sciences, University of Barcelona, 08907 Barcelona, Spain; 4 Service of the Medical-Surgical Area of Dentistry Hospital, University of Barcelona, 08907 Barcelona, Spain

**Keywords:** mandibular overdenture, early loading, dental implants, edentulous patients, geriatric patients

## Abstract

**Background**: This study aims to show the clinical outcomes of implants supporting mandibular overdentures in edentulous patients. **Methods**: Mandibular edentulous patients were diagnosed with an oral examination, panoramic radiograph, and diagnostic casts for intermaxillary relations and treated with overdentures over two implants. After two-stage surgery, implants were early loaded with an overdenture at 6 weeks. **Results:** Fifty-four patients (28 females and 24 males) were treated with 108 implants. Thirty-two patients (59.2%) had a previous history of periodontitis. Twenty-three patients (46%) were smokers. Forty patients (74.1%) suffered from systemic diseases (i.e., diabetes, cardiovascular diseases). The clinical follow-up of the study was 147.8 ± 10.4 months. The clinical outcomes showed a global success of 94.5% of implants. Fifty-four overdentures were placed in the patients over the implants. The mean marginal bone loss was 1.12 ± 0.34 mm. Nineteen patients (35.2%) showed some kind of mechanical prosthodontic complication. Sixteen implants (14.8%) were associated with peri-implantitis. **Conclusions:** Based on the clinical results obtained, we can determine that the treatment of elderly edentulous patients with mandibular overdentures through the early loading of two placed implants is a successful implant protocol.

## 1. Introduction

In geriatric edentulous patients, the use of conventional dentures has offered the benefit of providing improvement in function and esthetics. More recently, the introduction of implant-supported overdentures has become a better treatment option for edentulous patients. In fact, the implant-supported overdenture is considered as the first choice of standard care for edentulous patients because it offers a higher retention and stability provided by the attachment mechanism, opposing successful conventional maxillary dentures [1,2,3].

Geriatric edentulous patients with implant-retained overdentures were more satisfied with the comfort and mastication efficiency of their conventional dentures [4]. Implant-supported overdentures significantly improves stability and retention, oral function, esthetics, psychological well-being, and social functioning [5]. These aspects are endorsed, among other works, by the study by Egido-Moreno et al. [6]. In the mandible, the provision of two-implant overdentures will, in the majority of patients, significantly enhance levels of patient satisfaction [7]. 

During past decades, mandibular implant overdentures have been documented as an effective treatment for restoring the edentulous mandible [8,9,10]. Several studies have reported favorable clinical outcomes of implant treatment in geriatric edentulous patients, demonstrating that this treatment approach has a good implant survival [8,9,10]. The success rate of dental implants supporting overdentures is among the highest rates of implant dentistry. In fact, the majority of studies suggest that implant survival is greater than 95% [9,10].

The scientific evidence suggests that, for the rehabilitation of the edentulous mandible, a two-implant-retained mandibular overdenture should be the minimum standard of prosthetic care [2,9,11,12,13]. Different attachment types can be used for such overdentures, including bars of different designs, balls, and magnetic and resilient telescopic attachments. The Locator type attachment has become more popular as an attachment for unsplinted implants retaining overdentures because it provides easier hygiene, has a low vertical height, can be used with the inclination of the implants, involves low initial costs, and is compatible with implants from many different manufacturers [11,12,13].

Initially, for many edentulous patients treated with mandibular overdentures, a conventional loading protocol was established with an abutment connection after an initial healing period of 3–5 months [8,14]. Immediate loading protocol or early loading protocol for mandibular overdentures has been determined to be a well-established treatment and is worthy of consideration in clinical practice [15,16]. The decision to choose patients for immediate loading should be based on clinical parameters, bone quality, and primary stability of the implants placed. The recommendations for immediate or early loading were proposed with an initial insertion torque of 35 Ncm, or ISQ 60, via resonance frequency analysis (RFA) testing [17,18]. 

Although there are numerous studies that speak of the rehabilitation of patients through overdentures supported by two implants, this study aims to investigate the clinical results of the early loading of implants and long-term follow-up with mandibular overdentures in the treatment of edentulous patients.

## 2. Materials and Methods

This retrospective study was carried out at the Faculty of Dentistry at the University of Seville during the years 2008 to 2012. Due to the nature of clinical research, the principles described in the Declaration of Helsinki were taken into account for the design of the study, as well as the approval of the Ethics Committee of the University of Seville (Ethics Committee University of Seville-4-7-2012), and the informed consent of the patients were obtained [19].

**Figure 1:**
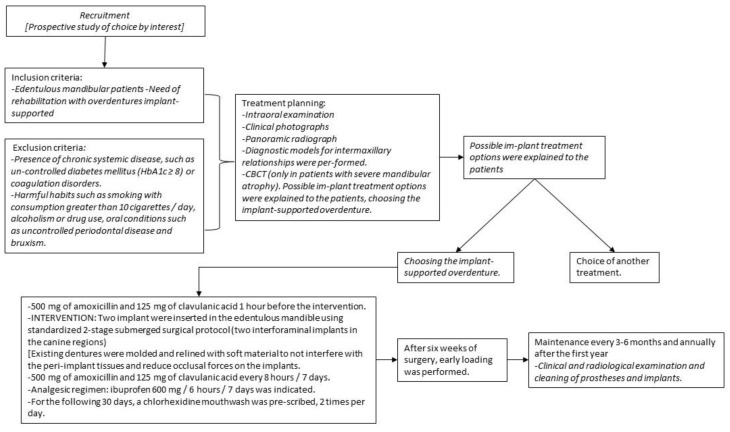
Study outline.

The inclusion criteria were edentulous mandibular patients in need of rehabilitation with implant-supported overdentures (Figure 1). The study population consisted of 54 patients, 25 women and 26 men, aged between 65 and 86 years, with a mean age of 72.5 years. It is a sample of convenience among patients who attend the University Clinic and choose an overdenture supported by two implants as treatment. The exclusion criteria were the following: (a) presence of chronic systemic disease, such as uncontrolled diabetes mellitus (HbA1c ≥ 8) or coagulation disorders; (b) harmful habits such as smoking with consumption greater than 10 cigarettes/day, alcoholism or drug use; and (c) oral conditions such as uncontrolled periodontal disease and bruxism. Regarding treatment planning, in addition to the intraoral examination and clinical photographs, a panoramic radiograph and diagnostic models for intermaxillary relationships were performed. In patients with severe mandibular atrophy, a CBCT was performed. Possible implant treatment options were explained to the patients who chose the implant-supported overdenture.

The patients were prescribed an antibiotic treatment consisting of 500 mg of amoxicillin and 125 mg of clavulanic acid 1 h before the intervention, as well as every 8 h/7 days after the treatment. As an analgesic regimen, ibuprofen 600 mg/6 h/7 days was indicated. For the following 30 days, a chlorhexidine mouthwash was prescribed to be used 2 times per day. Articaine with adrenaline as vasoconstrictor was injected as local anesthesia for implant treatment (Ultracaín Normon^®^, Barcelona, Spain: Articaine (INN)/Epinephrine (INN)). Hydrochloride of articaine, 40 mg/mL + 0.01 mg/mL solution was used for injection (1:100,000).

**Figure 2:**
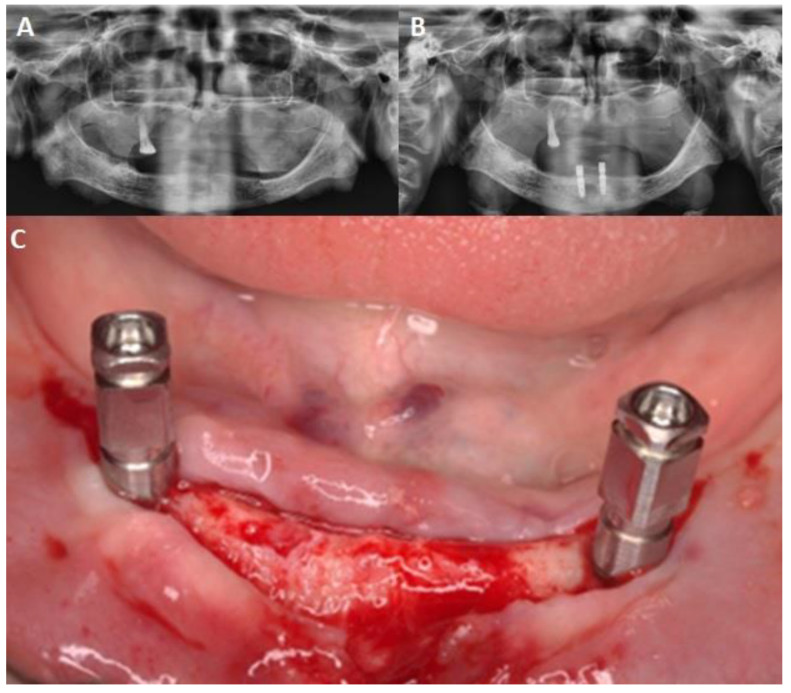
(**A**) Before orthopantomography; (**B**) post-orthopantomography; (**C**) inserted implants in edentulous mandible.

For all patients, two implants were inserted in the edentulous mandible using standardized 2-stage submerged surgical protocol (two interforaminal implants in the canine regions) (Figure 2). The CBCT was not routinely used based on socioeconomic factors and the date of the work. Implant surgery began with the preparation of two surgical beds, through the sequential steps of drills according to a protocol of progressive diameter increase. 

The implants selected for insertion in the patients were Surgimplant ^®^ (Galimplant, Sarria, Spain), which were characterized by an external connection and a surface treatment consisting of sandblasting and acid etching. Insertion torques were analyzed to determine implant stability after placement. An insertion torque ≥35 Ncm was considered adequate for implant stability at the time of placement [17,18].

**Figure 3:**
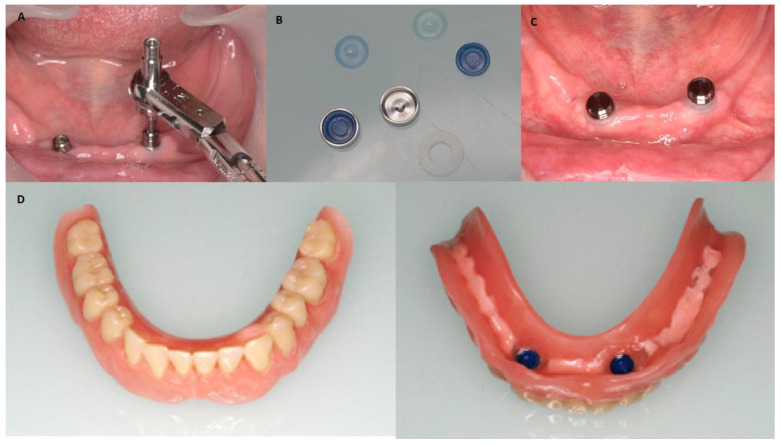
(**A**) Placement torque of prosthetic attachments (patrix). (**B**) Prosthetic attachments (matrix). (**C**) Placement of prosthetic attachments (matrix). (**D**) Overdenture with prosthetic attachments (matrix).

Existing dentures were molded and relined with soft material to avoid interference with the peri-implant tissues and reduce occlusal forces on the implants. Six weeks after surgery, early loading was performed. An open impression technique with individualized tray in addition to silicone material was used. An Overdent ^®^ (Galimplant, Sarria, Spain) telescopic attachment system was used in the manufacture and retention of the overdentures over the implants (Figure 3).

As criteria for success for implants, the following classic concepts were taken into account: stability and absence of mobility, absence of areas of radiographic radiolucency around the implant, absence of persistent and irreversible signs and symptoms (pain, suppuration, infection, paresthesia). The implants will be considered failed when they do not fulfill their purpose and have to be removed due to a lack of osseointegration or due to the presence of some infection, pain, or paresthesia [20,21]. All patients were included in a maintenance program consisting of clinical and radiological examination and cleaning of prostheses and implants. The frequency of revisions was set at 3 and 6 months and annually after guided implant insertion. Periapical radiographs acquired digitally using positioners were used for follow-up measurements of marginal bone loss. The analyzed variables included patient information (gender, age, dental health, systemic diseases, history of periodontitis, smoking habit), details about the placed implants (type, number, position, diameter, and length), and the implant-supported overdenture including the dates of delivery. In addition, surgical, biological, and technical complications that occurred during implant insertion, postoperatively, or during function in the follow-up period were recorded.

A statistical analysis of the variables obtained was carried out using SPSS software (SPSS^®^ 11.5.0, Chicago, IL, USA). Descriptive statistics were used to report the clinical results of the study. Absolute and relative percentage frequencies of qualitative variables were obtained, and chi-square test was used to analyze distributions. Means, standard deviations (SD), medians, ranges, and 95% confidence intervals (CI) were obtained for the quantitative variables, which were grouped by theme and frequency. An analysis of variance (ANOVA) was used to confirm the similarities in the groups. The analysis of differences between the groups created based on the different risk factors measured was performed using the non-parametric Mann–Whitney U test. The level of statistical significance was established for a value of *p* < 0.05.

## 3. Results

A total of 108 implants were inserted in 54 edentulous mandible patients, consisting of 26 males and 28 females. No significant statistical differences were found related to sex and age (chi-square test, *p* = 0.15008). Thirty-two patients (59.2%) had a previous history of periodontitis. Twenty-three patients (46%) were smokers (Table 1). A total of 40 patients (74.1%) exhibited systemic diseases (i.e., hypertension, diabetes), 24 patients with hypertension, 21 patients with diabetes, and 3 patients with other diseases. 

A total of 108 implants were inserted in 54 patients. Fifty-seven implants (52.8%) had a diameter of 4 mm, and fifty-one implants (47.2%) had a diameter of 3.5 mm. A total of 56 implants (51.8%) were 10 mm in length, and 52 implants (48.2%) were 12 mm. Six implants (5.5%) were lost during the follow-up (Table 2). In two patients, both implants were lost during the early period of osseointegration; in one patient, the implants were lost at 2 months; and in the other patient, the implants were lost at 3 months. Additionally, in two patients, an implant was lost, one at 18 months and the other at 22 months. All implants were successfully replaced. The average follow-up period was 147.8 ± 10.4 months (range of 120–169 months). The cumulative survival rate for all implants was 94.5%. There were no differences between the different antagonists in this study.

The mean marginal bone loss was 1.12 ± 0.34 mm, ranging from 0.7 to 1.85 mm during the follow-up evaluation (Table 3). In patients less than 75 years of age, the marginal bone loss was 1.02 ± 0.21 mm compared with 1.10 ± 0.25 for more than 75 years of age (ANOVA; *p* = 0.0667). On the other hand, in female patients, the marginal bone loss was 1.05 ± 0.28, compared with 1.19 ± 0.30 in male patients (ANOVA; *p* = 0.5793). In patients with a history of periodontitis, the marginal bone loss was 1.30 ± 0.24, compared with 1.12 ± 0.26 in patients without a history of periodontitis (ANOVA; *p* = 0.0762). In patients who smoke, the marginal bone loss was 1.16 ± 0.27, while the marginal bone loss in patients without smoking habits was 1.08 ± 0.26 mm (ANOVA; *p* = 0.3055). Referring to patients with systemic diseases, the marginal bone loss was 1.12 ± 0.22, and 1.11 ± 0.31 for patients without medical conditions (ANOVA; *p* = 0.9259). Finally, in patients with a follow-up of less than 150 months, the marginal bone loss was 1.05 ± 0.27, compared with 1.13 ± 0.23 with a follow-up of more than 150 months, with statistical differences (ANOVA; *p* = 0.5992).

During the follow-up period, 16 implants (14.8%) in 12 patients (22.2%) were associated with peri-implantitis (Table 4). Peri-implantitis was more frequent in those patients with a previous history of periodontitis (41.6%) and in patients who smoke (33.3%). 

After a 6-week healing period, 54 overdentures were performed for over 108 implants placed in the patients. Technical prosthodontic complications were recorded in 19 patients (35.2%) (Table 4). Seventeen patients (31.5%) needed to change the locator-attachment system, and six patients (11.1%) showed resin fracture of a prosthesis.

## 4. Discussion

The present study evaluated the clinical outcomes in geriatric edentulous patients treated with mandibular overdentures by the early loading of two implants. The clinical outcomes demonstrated that implant-supported mandibular overdentures are a good alternative for restorative solutions of edentulous patients with a cumulative survival rate for implants of 94.5% after a 12-year follow-up. The most frequently observed complications were mechanical (35.2%) and biological complications (22.2%).

Several studies reported the long-term outcomes of implant-supported mandibular overdentures with high implant survival rates [7,13,22,23,24]. A clinical retrospective study of 495 patients confirms the longevity of implants in the overdenture therapy for the mandible with a cumulative survival rate for the supporting implants of above 95% after 23 years of loading. From the 1051 inserted implants, 41 failed (3.9%), including 4 before the abutment connection (0.4%), 24 soon after the abutment connection (2.3%), and 13 after loading (1.2%) [22]. A prospective study reported the outcomes of 150 edentulous patients with two implants to support a mandibular overdenture. The clinical and radiographic parameters that were assessed after 10 years of functional loading had a survival rate of 95.3% (91.4% for IMZ implants, 98.3% for Branemark implants, and 99% for ITI implants) [23]. A long-term prospective study on mandibular overdentures supported by two implants and a bar-clip attachment in an elderly population (20-year follow-up) showed a high implant survival of 92.5% [24].

Initially, the delayed loading protocol proposed that the implants should be left submerged and unloaded for a period of 3 to 6 months to facilitate osseointegration, avoid soft tissue encapsulation, and improve a high implant survival rate [25]. However, clinical and experimental studies have demonstrated that osseointegration can be achieved with immediate and early loading protocols [26,27]. The clinical outcomes of the early loading of implants supporting mandibular overdentures have compared with delayed and immediate loading protocols with similar results that show a high success rate [28,29]. The early loading protocols for splinted implants supporting mandibular overdentures have been previously proposed in initial studies, particularly developed by the basis of the use of roughened titanium surfaces [15,30]. The early loading of implants for mandibular overdentures (between 1 week and 2 months) has been recommended without impairing the implant success rate [2,9,15,17,18,31]. A prospective study reports the clinical results of 15 consecutively treated patients by an early loading protocol using two implants with a mandibular overdenture supported by a resilient ovoid bar mechanism. The patients were followed for an average of 28.87 ± 5.04 months. The overall success rate was 100% for the implants and 93% for the prosthetic treatment [31]. A recent study reported the clinical outcomes of 14 mandibular edentulous patients treated with overdentures over two implants by guided surgery, and early loading at 6 weeks [2]. The clinical follow-up of the study was 44.7 ± 31.4 months. The clinical outcomes showed a global success of 100% of implants, indicating that this early loading protocol appears to be a successful implant treatment [2]. 

Various types of implant-supported overdentures for mandibular edentulism are reported in the literature [2,7,8,12,13,14,15]. In the present study, all patients were treated with an overdenture retained by a Locator^®^ type attachment that was connected to two implants. The question of whether there is an optimal number of implants in mandibular implant-retained or supported overdentures has been raised for several years [1,32]. Most recently, the literature reported no significant differences between two implants with ball or bar attachments or four interconnected implants. The four-implant bar treatment provided greater prosthesis retention than the other treatment types, but after experience with different systems, the two-implant supported overdenture is the ideal prosthesis, because it is retentive, and appears to be less costly, less technique sensitive, and more accommodating of anatomical arches [32]. Today, there is overwhelming evidence to support the proposal that a two-implant overdenture should become the first choice of treatment for the edentulous mandible [1,6,32]. 

Mandibular two-implant-retained overdentures are considered a reliable treatment option for the rehabilitation of geriatric edentulous patients [11,12]. Mandibular overdentures are connected to the implants using different attachment systems. In order to derive satisfactory retention for the patient, various attachments were employed, including bar, ball, stud, and magnet attachments. The retentive force of the attachments is obtained via mechanical interlocking, frictional contact, or magnetic forces [11,12]. Retention by Locator^®^ type attachments is a widely used system for implant-supported or implant-retained overdentures that improves stability and appropriate retention [2,12,33]. It offers interchangeable plastic inserts that are available in different retention values. The use of this attachment can have a positive effect on the oral health quality of life of the patients treated with mandibular overdentures [12,33]. However, this attachment reports extensive deformation and requires considerable maintenance with the replacement of small plastic matrices, but has a low cost [34].

The evaluation of the marginal bone loss is an important standard for implant success [3,6,24,35,36,37]. Different studies reported data on the marginal bone loss of the long-term outcome of implants supporting a mandibular overdenture [6,24,36,37]. The factors that jeopardize these clinical outcomes include smoking, history of periodontitis, bone quantity, and an increased follow-up period [6,33,34,35]. In the present study, the mean marginal bone loss was 1.12 ± 0.34 mm. The marginal bone loss was higher in smokers and in patients with a background of periodontitis. We are aware of the bias that setting the limit of 10 cigarettes per day in smokers may represent, an aspect that is also considered by other authors [38,39]. These clinical findings are confirmed by several studies [2,36,40]. In patients treated with mandibular overdentures with a mean follow-up period of 44.7 ± 31.4 months, with a history of periodontitis, the marginal bone loss was 1.40 ± 1.10 compared with 1.19 ± 0.88 in patients without a history of periodontitis [2]. The effect of cigarette smoking on the peri-implant tissues was confirmed in a 16-year retrospective study of two implant-supported overdentures that shows an increased marginal bone loss in smokers [36]. Although in our study, the marginal bone loss in women is somewhat greater than in men, it is not significant; similar results were obtained in other studies by our group and coincide with recent reviews that do not include sex as a risk factor of peri-implantitis [40,41,42]. A prospective study reports the clinical outcomes of mandibular overdentures for 20 years. The marginal bone loss was 0.35 mm greater at 20 years than at 10 years, that is, 0.04 mm per annum between 10 and 20 years with a range from a loss of 1.35 mm to a gain of 0.37 mm [37]. Moreover, different types of overdenture attachments and loading protocols (delayed, early, and immediate) had a similar effect on the marginal bone loss. The scientific evidence suggests that the marginal bone loss around inserted implants in mandibular overdentures with delayed, early, and immediate loading do not reveal differences. Similar results about the marginal bone loss are observed according to the Locator attachments or the ball anchors [3,17,43,44].

Biological problems as mucositis and peri-implantitis of infectious inflammatory etiology can affect the peri-implant tissues. The risk factors for peri-implant diseases includes smoking habits, a history of periodontal disease, inadequate maintenance attendance, and neglected oral hygiene [45]. These biological complications can occur in edentulous patients treated with a mandibular overdenture supported with implants [2,8,23,46,47]. In the present study, 16 implants (14.8%) in 12 patients (22.2%) were associated with peri-implantitis. One aspect that can explain the low rates of peri-implantitis found is the surface of the implants used (Nanoblast plus^®^, Galimplant, Sarria, Spain). It is a surface obtained by coarse-grained sandblasting and subsequent triple acid etching, resulting in a surface that is free of aluminum, free of ions, and free of other types of impurities. There are various works, some recent [48,49], which report that the surface of ultrahydrophilic and nano-structured dental implants can influence the long-term outcome, including survival, success rates, and the development of complications related to the implant, including peri-implantitis. Peri-implantitis was more frequent in those patients with a previous history of periodontitis (41.6%) and in patients who smoke (33.3%). A retrospective study reported a good peri-implant issue health as the success criteria of two implant-supported mandibular overdentures. A total of 43 patients with 86 implants were included with an average observation period of 41.8 months. No implants showed peri-implantitis, and only eight implants (9.3%) showed peri-implant mucositis [46]. Meijer et al. [23] reported an incidence of peri-implant mucositis in patients treated with mandibular overdentures of 51.9% after 5 years of evaluation, and 57.0% after 10 years. The incidence of peri-implantitis was 16.9% after 5 years of evaluation, and 29.7% after 10 years [23]. Rinke et al. [47], in a retrospective study, reported peri-implantitis in 37.5% of a total of 24 mandibular overdentures. The prevalence of peri-implantitis (radiographic bone loss ≥3.5 mm) was evaluated via a digital analysis of panoramic radiographs taken postoperatively and after 5–19 years of clinical function. The mean observational time was 7.3 years. The prevalence of peri-implantitis was more frequent in patients who smoke [47].

The maintenance requirements and the presence of prosthetic complications associated with the implant-retained overdenture must be evaluated [35,50,51]. Mechanical complications were frequent in the present study. Nineteen patients (35.2%) showed some kind of technical complication. Seventeen patients (31.5%) needed to change the locator attachment system, and six patients (11.1%) showed resin fracture of a prosthesis. These clinical findings are confirmed by a 5-year study of the prosthetic complications and maintenance of different attachments used to stabilize mandibular two-implant overdentures [45]. The majority of prosthetic complications occurred in the first year. The most frequent prosthetic complication was wear/distortion of the retentive components of the locator and telescopic attachments and the activation of the clips of the bar attachment [51]. The initial retention force of the attachment system is high. The matrix resiliency among different attachment systems can be compromised by the insertion and removal of the overdenture [12,34]. The retention loss, related to the wear of the retention device, is in common need of maintenance, requiring activation or replacement that can be easily provided by the clinician in clinical practice [35,50]. This event affects all patients, and it can occur several times during the follow-up. The metallic attachment can be activated (gold alloy) or replaced (plastic cap, nylon, or rubber ring). According to clinical experience, this procedure is considered successful if there are no more than two activations, repairs, or replacements of either component in the first year of clinical use [52,53,54]. 

The difference in the failure found between men and women is a controversial issue, which some authors attribute to the greater masticatory strength of men [54], but which not all share [55].

The present study has some limitations, such as the fact that it is a retrospective study, and the implants were of different lengths and thicknesses, which are aspects that were considered to be advantages at the same time. Another limitation of the study is that there are no exact data on the level of the hygiene of the patients, but it should be considered that the clinical group performs annual maintenance on the patients.

## 5. Conclusions

Implant dentistry is widely used as a comprehensive alternative to planning for surgical and prosthodontic steps for the treatment of geriatric edentulous patients. The clinical results obtained in this study indicate that the treatment of elderly edentulous patients with mandibular overdentures by the early loading of two implants appears to be a successful implant protocol.

## Figures and Tables

**Table 1:** Description of patients’ characteristics.

	*n*	%
Males	26	48.1
Females	28	51.9
History of periodontitis	32	59.2
Smokers *	23	42.6
Systemic diseases	40	74.1

*n* = patient. * Consumption of more than 10 cigarettes/day.

**Table 2:** Description of implant characteristics.

	*n*	%
4 mm implant diameter	57	52.8
3.5 mm implant diameter	51	47.2
10 mm implant length	56	51.8
12 mm implant length	52	48.2
Loss of implant	6	5.5

*n* = implant.

**Table 3:** Mean marginal bone loss of patients.

Variable			*p* Value
Age *	≤75 yr	>75 yr	
	1.12 ± 0.21	1.10 ± 0.25	*p* = 0.0667
Gender	Female	Male	
	1.05 ± 0.28	1.19 ± 0.30	*p* = 0. 5793
History of periodontitis	+	-	
	1.30 ± 0.24	1.12 ± 0.26	*p* = 0.762
Smokers *	+	-	
	1.16 ± 0.27	1.08 ± 0.26	*p* = 0.3055
Systemic diseases	+	-	
	1.12 ± 0.22	1.11 ± 0.31	*p* = 0.9259
Follow-up *	≤150 months	>150 months	
	1.05 ± 0.27	1.13 ± 0.23	*p* = 0.5992
Total	1.12 ± 0.34 (0.7–1.85)		

* Consumption of more than 10 cigarettes/day.

**Table 4:** Description of patients with complications.

	*n*	%
Implant loss	6	11.1
Peri-implantitis	12	22.2
Technical complications	19	35.2

## Data Availability

Not applicable.
